# Using Omics Technologies and Systems Biology to Identify Epitope Targets for the Development of Monoclonal Antibodies Against Antibiotic-Resistant Bacteria

**DOI:** 10.3389/fimmu.2019.02841

**Published:** 2019-12-10

**Authors:** Antonio J. Martín-Galiano, Michael J. McConnell

**Affiliations:** Intrahospital Infections Laboratory, National Centre for Microbiology, Instituto de Salud Carlos III, Majadahonda, Spain

**Keywords:** monoclonal antibodies, antibiotic resistance, multidrug resistance, systems biology, big data, immunoinformatics, bound rationality

## Abstract

Over the past few decades, antimicrobial resistance has emerged as an important threat to public health due to the global dissemination of multidrug-resistant strains from several bacterial species. This worrisome trend, in addition to the paucity of new antibiotics with novel mechanisms of action in the development pipeline, warrants the development of non-antimicrobial approaches to combating infection caused by these isolates. Monoclonal antibodies (mAbs) have emerged as highly effective molecules for the treatment of multiple diseases. However, in spite of the fact that antibodies play an important role in protective immunity against bacteria, only three mAb therapies have been approved for clinical use in the treatment of bacterial infections. In the present review, we briefly outline the therapeutic potential of mAbs in the treatment of bacterial diseases and discuss how their development can be facilitated when assisted by “omics” technologies and interpreted under a systems biology paradigm. Specifically, methods employing large genomic, transcriptomic, structural, and proteomic datasets allow for the rational identification of epitopes. Ideally, these include those that are present in the majority of circulating isolates, highly conserved at the amino acid level, surface-exposed, located on antigens essential for virulence, and expressed during critical stages of infection. Therefore, these knowledge-based approaches can contribute to the identification of high-value epitopes for the development of effective mAbs against challenging bacterial clones.

## Do We Need Monoclonal Antibodies Against Antibiotic-Resistant Bacteria?

In recent years, there has been an explosive increase in the emergence and dissemination of antimicrobial resistance. Multiple factors have likely contributed to this phenomenon, including the overuse of existing antibiotics in the clinical setting, non-human use of antibiotics, and increased international travel. A report commissioned by the United Kingdom in 2014 estimated that deaths directly attributable to antimicrobial resistance will increase to 10 million annually by 2050, as compared to the 700,000 deaths currently produced by these infections per year (1). The predicted economic expense caused by antimicrobial resistance is also significant, as the same study projected that the cumulative worldwide loss of Gross Domestic Product (GDP) between 2014 and 2050 would be higher than the current yearly GDP of all countries combined. Although these extreme scenarios represent projections based on current trends, there is little doubt that antimicrobial resistance will be a major public health threat in the near future. Importantly, the clinical management of multidrug-resistant (MDR) infections is complicated by the lack of currently approved antimicrobials that retain sufficient activity against MDR strains, particularly the so-called ESKAPE microorganisms, which include *Enterococcus faecium, Staphylococcus aureus, Klebsiella pneumoniae, Acinetobacter baumannii, Pseudomonas aeruginosa*, and *Enterobacter* spp. (2). Recent sporadic reports from different geographic regions describing pan-drug resistant isolates, with resistance to all clinically-available antibiotics, are cause for particular concern (3–5). In this context, the need to develop new antibiotics, ideally with novel mechanisms of action not affected by cross-resistance to existing mechanisms, is apparent. Unfortunately, while there have been recent approvals of new antibiotics for clinical use, very few antimicrobials with completely novel mechanisms of action have been developed over the last 40 years.

While new antibiotics will be key players in combating resistance, it is likely that treatment and prevention approaches fighting on alternative fronts will need to be explored. In this regard, a recent report summarizing the portfolio of alternatives to antibiotics that are currently under development identified antibody-based therapies, probiotics, phage therapy, immune stimulation, and vaccines as “Tier 1,” based on their stage of development and probability of success (6). Among these approaches, therapies based on monoclonal antibodies (mAbs) have a number of characteristics that may make them ideally suited for the treatment and prevention of infections caused by MDR bacteria, including (a) absence of susceptibility to existing resistance mechanisms and lack of selection for resistance to existing antibiotics, (b) facilitating immune-mediated clearance of bacterial pathogens, (c) high specificity and therefore minimal effects on non-target bacteria present in the human microbiota, (d) safety and efficiency in humans, and (e) passive immunization, which, in contrast to active immunization with vaccines, has potential to provide immediate protective immunity against infection, which may be particularly important in critically-ill patients with decreased immune function. In this review, we assess the potential of omics technologies and systems biology approaches to enhance the rational identification of epitopes for the development of mAbs against MDR bacteria.

## Challenges to Developing mAbs For Resistant Bacteria

MAbs are highly directed therapeutics that embody the magic bullet ideal of specifically targeting a particular pathogen. However, despite the fact that a large number of therapeutic mAbs have been successfully developed for multiple different human pathologies, most notably for rheumatologic and oncologic diseases, only three mAb therapies have been approved for bacterial infections. Raxibacumab and obiltoxaximab have been developed for inhalational anthrax (7, 8), while bezlotoxumab was recently approved for the prevention of *Clostridium difficile* infection (9). The relative paucity of mAbs for bacterial infections is especially noteworthy given the key role played by antibodies in bacterial clearance during natural infection and vaccine-induced immunity. However, the difference in the rate of increase in approved antibodies for different disease types may be partially due to the fact that the features of the underlying biology being targeted by mAbs for non-infectious diseases are very different from those in pathogenic bacteria. In the former cases, highly conserved human proteins, either cancer antigens or immune effector molecules (e.g., cytokines), are targeted. In stark contrast, antibacterial mAbs target rapidly dividing microorganisms with high genetic plasticity. Bacteria have the ability to downregulate or even completely abolish the expression of molecules containing targeted epitopes, in a process generally known as epitope masking (10). Moreover, these microorganisms can exert epitope switching since they are able to modify and tolerate severe amino acid changes in epitopes that reduce antibody affinity through recombination with externally-acquired DNA or via mutations that do not produce significant changes in virulence and fitness (11, 12). This is a consequence of confronting double Darwinian pressure in search of an equilibrium between keeping important functions for (patho)biology and the evasion of host immunity. By doing so, bacterial pathogens have evolved to avoid detection and neutralization by antibodies.

In the three aforementioned antibacterial mAbs, disease is prevented due to the neutralization of toxins via binding to highly conserved epitopes on toxin subunits. This approach is effective for anthrax and *C. difficile* infection due to the fact that these pathologies are mediated by the action of potent toxins. However, this is not the case for most bacterial pathogens, particularly for MDR-associated species. MAbs for these infections will most likely need to target epitopes present on the bacterial cell and facilitate clearance by the immune system to be effective. In this regard, bacterial epitopes that would be ideal targets for mAbs may need to meet most, if not all, of the following criteria: (a) high conservation between circulating strains, (b) expression during bacterial infection and/or colonization, (c) surface exposure in order to permit antibody binding, and (d) antigenically distinct compared to epitopes on human proteins and the normal human microbiota to prevent cross-reactivity.

The biological function of the molecule containing the targeted epitope may be of particular importance. MAbs that target epitopes on molecules that participate in essential bacterial processes for viability or virulence may be less susceptible to the generation of escape mutants, given that reduced expression or sequence variation in these molecules may be detrimental to bacterial survival. It is worth noting that MDR organisms consist of a series of interacting molecular elements, a functional network, with emergent properties only approachable as a whole by systems biology and high-throughput “omics” techniques (13). One of the emergent properties of scale-free networks is the tolerance to failures that, in this case, means that many essential bacterial processes are subject to total or partial functional redundancy (14). Bacteria can increase fitness mainly by gathering genes that exert functions efficiently but that can be, at least partially, covered by other means. For example, 30 alternative sugar transporters (15) and seven plasminogen-binding proteins have been identified in *Streptococcus pneumoniae* (16) that ensure that these important tasks required for infection are performed under many conditions. Likewise, antigenic proteins rarely act in isolation but rather as part of functional sub-networks that exert simultaneous or sequential activities leading to colonization and/or disease (13). In this respect, epitope switching by mutation or down-regulation of a single participant targeted by mAbs in one of these pathways may not greatly affect the ability of the bacteria to replicate and produce infection if this change can be adequately compensated for elsewhere in the interactome. Thus, non-overlapping irreplaceable elements of the pathofunctional sub-networks, i.e., virulence hubs, should be prioritized. This may present particular challenges in identifying a single epitope for mAb development that is less susceptible to the generation of escape mutants.

## Using Omics Technology and Systems Biology For mAb Development

### Omics Data and Systems Biology Basics

Although there are significant challenges to developing broadly effective mAb-based therapies for bacterial infections, it is conceivable that the availability of multiple large data sets involving genomic sequences and global profiling experiments (e.g., transcriptomics, proteomics, and interactomics; the latter defined as the global pool of physical and/or functional connections between molecules in a cell) may serve as raw material for elucidating high-value epitopes. Table 1 lists the different omics approaches that are discussed in the sections below and how they can be employed for mAb development. Omics technologies can be considered as those that characterize molecules and their states at a holistic level through the collective characterization of molecular profiles, e.g., transcriptomics, the whole set of transcripts under defined conditions. Omics cover virtually all kinds of biological molecules, and their accumulated outcome volumes approximate to the range of big data, i.e., so massive that they are unable to be stored and managed by ordinary computer users. For instance, central repositories such as the European Bioinformatics Institute store over 160 petabytes of data (17).

**Table 1:** Use of omics technologies and systems biology in antibacterial mAb development.

**Technology**	**Use in identifying epitopes/antigens**
Comparative genomics	- Identification of epitopes with highly conserved sequences - Identification of epitopes present in the majority of strains within a species - Clonal distribution of epitopes - Avoidance of cross-reaction with microbiota and human proteins
Transcriptomics	- Identification of antigens preferentially expressed during infection
Proteomics	- Identification of antigens highly expressed during infection - Identification of epitopes on the bacterial cell surface
Molecular modeling and dynamics	- Identification of surface-exposed epitopes - Assessment of the stability of surface exposure of the epitope and its binding to the antigen
Interactomics/systems biology	- Identification of optimal synergistic mixtures of epitopes (for use in developing mAb cocktails) - Identification of epitopes/antigens that participate in essential bacterial processes that involve molecular connections

While processing large biological data sets could be considered mere brute force, it is important to underscore that the data needed for translational medicine are those that contribute to achieving precise clinical goals, so-called “smart data” (18). Systems biology approaches move in that direction by permitting a more comprehensive and contextual interpretation of the information, given that they can identify not only epitopes meeting certain criteria but also the interplay between different essential criteria (19). The integration of multivariate data for rational vaccine purposes is far from trivial (20); clearly the challenge here is converting large quantities of data into information with biological value that can be used for the development of mAb therapeutics. As data volumes become larger and more varied due to the availability of multi-omics experiments, it is here that systems biology can be of great value in responding to biological problems of great complexity. Systems biology then becomes a natural analysis option that captures emergent properties of bacteria as a whole that cannot be studied by isolated reductionist protocols. The complexity of the immune response to vaccines has been monitored and analyzed through an analogous approach called systems vaccinology, a branch of systems immunology concentrated on the intrinsic responses of the host to vaccines (21). Parallels can be drawn to reverse vaccinology (RV), which employs both genomics and structural biology to reveal the fraction of the molecular space of a pathogen appropriate for vaccine development (22). It is noteworthy that RV has already yielded successes, most notably in the development of a vaccine for *Neisseria meningitidis* (23). In contrast to RV for vaccines, which can operate at the antigen level, omics and computational approaches for mAb development must be performed at the epitope level, potentially adding increased stringency and complexity to the identification process. Thus, RV is confronted with a significant challenge in the design of mAbs, and the question arises of whether such obstacles hamper knowledge-based solutions. This situation evokes the “bounded rationality” idea (24, 25), in which rational approaches are inefficient due to limited understanding of the inherent complexity of the task.

In the following sections, we assess how the availability of huge and variable data sets can be harnessed, together with systems biology, to enhance RV when oriented to epitope selection for mAb development.

### Comparative Genomics: Whole Species and Clone-Specific Epitope Conservation

**Figure 1:**
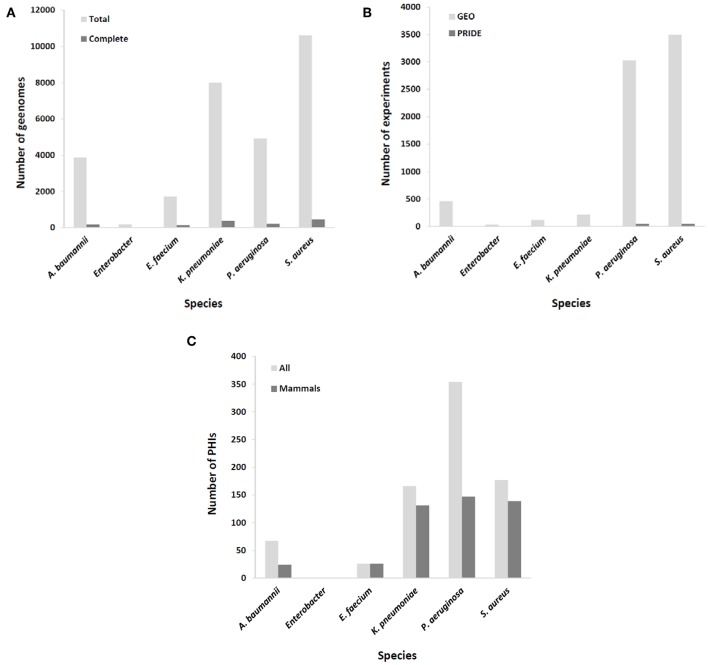
Coverage of ESKAPE organisms by omics databases utilized in rational mAb development. **(A)** Number of available complete and draft/scaffold genomes; **(B)** number of expression experiments, either transcriptomic (GEO database) or proteomic (PRIDE database); **(C)** number of identified molecular interactions between pathogen and host (total and those involving only mammal hosts).

A common challenge in developing immunoprotective approaches for bacterial infections is that these microorganisms exhibit a high rate of escape from vaccine formulations at the whole species level. Thus, the ideal of identifying immutable antigenic proteins as part of the core proteome of target species and absent from other species is difficult to achieve and may be confined to the aforementioned exotoxins that have already been exploited for mAb development (7–9). Nevertheless, the availability of up to thousands of draft genome sequences for the most important MDR pathogens may enable the assessment of epitope conservation at intra-clonal resolution with sufficient depth (Figure 1A). A plausible strategy may be to focus on the development of mAbs tailored to circulating hypervirulent and/or hyperresistant clones for which the recognized epitope is conserved. This is, for instance, the case for mAbs directed against *K. pneumoniae* O-antigen from the ST258 clone, since it is a recurrent infective lineage and a strong producer of this endotoxin (26). In addition, such clonal specificity preserves the microbiota, highlighting one of the advantages of immunological interventions with respect to antibiotics (27).

### Identifying Epitopes Highly Expressed During Infection and/or Colonization

Epitopes of interest for mAb development must be expressed during the course of colonization or infection. Rather than constitutive, the expression of many immunogenic proteins is tightly repressed in order to reduce metabolic expense and overexposure to the host immune system, unless the bacterium senses the right environmental signals for its production (28). To include this important issue in the mAb production pipeline, results from a number of transcriptomic and proteomic experiments stored in databases such as GEO and PRIDE (29), respectively, should be taken into account. MDR microorganisms are well-covered in this respect (Figure 1B), including the upregulation of potentially antigenic proteins for mAbs *in vivo* or under *in vitro* conditions that mimic infection, such as bacteremia (30), biofilm (sessile)-to-planktonic transition (31), and iron limitation (32).

### Exposure of the Epitope

MAbs must not only fulfill the basic RV principle of being directed against surface or secreted proteins but must also be directed toward epitopes that are exposed on these proteins. This poses a problem on two levels. First, accessible—either secreted or surface—proteins can be detected by the presence of motifs and domains linked to secretion and surface anchoring, in most cases readily detectable by sensitive hidden-Markov models thanks to optimized heuristics adapted to huge protein datasets (33). However, these computational strategies cannot cope with accessible proteins lacking identifiable labels, and must therefore be complemented with experimental high-throughput protein detection on fractionated samples, including cell-free medium for the exoproteome (34) or the outer membrane (35) and cell wall (36) for the surface proteome. On the other hand, the prediction of non-linear epitopes and their location on the solvent-oriented zone of the protein is facilitated by structural information (37). Resolving a structure is labor-intensive, but the combined effort of small scientific groups and large structural genomics consortia (38) has promoted the inclusion of structural biology to the biological pool of large data volumes. The central structural repository, the Protein Data Bank (PDB), currently contains 142,433 proteins (44,971 non-redundant, last accessed: 05/Jul/2019). Likewise, the SwissModel archive reached 1.6 million pre-built structural models (39) covering 62% of *S. aureus* and 72% of *P. aeruginosa* proteins. Once reliable structural information is available for the candidate antigen, epitopes that are highly solvent-accessible can be identified. Dynamic simulations by simulator packages such as GROMACS additionally permit assessment of the dynamic stability of epitope exposure and even of mAb-protein binding when co-crystalized (40).

### Design of Anti-virulence mAbs Using Functional Information

As a rational approach, functional information regarding the essentiality of a protein carrying the candidate mAb epitope or its involvement in virulence is invaluable. If these essential/virulence-associated epitopes are not targeted, there is high risk of epitope masking or switching, leading to rapid circumvention of the monoclonal therapy. Specialized resources such as PATRIC, VFDB, and Victors (41–43) compile virulence factors at the species level from dedicated research reports. Laboratory and animal-model screenings, such as signature-tagged mutagenesis (44), permit the explicit detection of potential genes essential for pathogenesis in a high-throughput manner. Such relevant information would be a promising starting point for antigen selection and prioritization to block virulence traits with mAbs in a precise knowledge-based way. Considering that most virulence factors are not essential for fundamental viability, neutralization by mAbs is akin to blocking virulence rather than the viability of the pathogen and follows the anti-virulence drug paradigm, in contrast to lethal antibiotics (45). Currently licensed mAbs that block the activity of exotoxins can be considered virulence-blocking therapies.

### Interactomics

According to systems biology principles, the pathogen and host exhibit a dense network of inter-species molecular interactions throughout their relationship (46). The identification of these interactions has permitted the design of protective strategies in viruses (47). This information may be used to design mAbs that impede connections between pathogen and host molecules that are central to infection progression. A proficient resource for this information is the PHI-base database (48), but of the 12,466 interactions included (Last accessed, 5 Sep 2019), only 467 pertain to connections between the six ESKAPE bacteria and mammals, suggesting that the volume of useful information could still be increased to facilitate the prediction of network tolerance in a global manner (Figure 1C). A possible exception is those pathogens whose virulence is almost fully dependent upon the activity of potent exotoxins, which indicates that successful mAb strategies at present are those that circumvent the pitfall of pathogenic network tolerance.

## Omics-Systems Biology vs. Other Strategies

A fundamental controversy may arise when systemic computational approaches in the RV framework are compared to empirical screenings (49) or the low-throughput selection of antigen/epitope targets (50) by microbiology experts. Each of these strategies has advantages and pitfalls based on their underlying assumptions (Table 2), but it is worth noting that the only mAb products that have been approved or are in the clinical phases of testing were developed using the latter two approaches. This may call into question the use of rational approaches based on RV strategies for identifying epitopes for mAb development. More empirical approaches may have achieved their previous successes because experimental screenings are better equipped to accommodate the degenerate and flexible nature of the immune system. Nevertheless, massive data and systemic approaches have likely not yet been successful, not because of their lack of potential, but because of the interpretation of results (51, 52). In addition, there is room for improvement for rational approaches through the collection of new data, algorithms, and paradigms, and this is a continuous process, whereas screening and expert selection are probably closer to their respective plateaus. Moreover, different methods of omics data integration can be developed in order to identify targets for biomedical applications (13). Conceivably, hybrid approaches that combine the strengths of all of these methods may achieve the highest performances. For instance, a list of the candidate mAb epitopes that have a complex list of features could be revealed from large data sets, verified by experts, and then refined by screenings methods.

**Table 2:** Pros and cons of systems biology/big data/reverse vaccinology approaches vs. empirical screening vs. expert selection for mAb development.

**Aspects related to mAb development**	**Omics/systems biology**	**Empirical screening**	**Expert selection**
Use for mAb cocktail development	++	–	–
Reduced cost	+	–	++
Time required	+	–	++
Focus on clinical clones	++	+	+
Requires bioinformatics expertise	–	++	++
Requires computational infrastructure	–	++	++
Requires experimental infrastructure	++	–	+
Intrinsic experimental validation	–	++	++
Rational selection	++	–	++
Resistance to “bound rationality”	–	++	+
Room for improvement	++	+	+
Scalability to many targets	++	+	–
Species completeness	++	+	+
Systemic view	++	–	–
Transferability to other species	++	–	–

*++ Highly efficient*.

*+ Moderately efficient*.

*– Low efficiency*.

An option that may also ease the “bound rationality” of RV is the design of mAb cocktails. The advantages of increasing valence by using mAb combinations are multiple: (1) a net increment in the success rate of neutralization of a process by reducing the network tolerance of the pathogen; (2) lower chances of future immune evasion since several concerted epitope switches are exponentially more difficult to achieve than individual ones; (3) the possibility of designing complex blocking strategies concentrated on the same (pathogen siege) or sequential (pathogen exhaustion) stages of infection, thus applying a more comprehensive molecular view of the virulent process. Knowing this, the bottleneck in mAb development against bacteria may not lie in the experimental efficiency of mAb identification but in scaling processes required for the production of a mature pharmaceutical product.

## Conclusions and Future Directions

In contrast to cancer, rheumatologic diseases, and viral infections, the limited use of mAbs for MDR bacterial pathogens may be due to several technical and biological constraints. In this context, rational approaches based on large-scale data/systems biology methodologies may facilitate the identification of high-value epitopes for mAb development, perhaps in concert with traditionally used empirical strategies. The extreme challenge associated with finding ideal, immutable epitopes may support the development of mAb cocktails. This could require improvement in the efficiency (development and scaling) of mAb production, i.e., within a reasonable timeframe and at a reasonable cost, at the service of holistic paradigms that consider the molecular pathobiology of the targeted species. We envisage that the large data/systems biology combination will find its utility in RV approaches applied to mAb development as more information is collected.

## Author Contributions

MM and AM-G planned the manuscript content, wrote the manuscript, and approved the final version.

### Conflict of Interest

MM is a founding partner and shareholder in Vaxdyn, a biotechnology spin-off company developing vaccines and antibody-based therapies for antibiotic resistant infections caused by antibiotic resistant bacteria. The remaining author declares that the research was conducted in the absence of any commercial or financial relationships that could be construed as a potential conflict of interest.
